# Minimal Residual Disease Monitoring with Next-Generation Sequencing Methodologies in Hematological Malignancies

**DOI:** 10.3390/ijms20112832

**Published:** 2019-06-10

**Authors:** Ricardo Sánchez, Rosa Ayala, Joaquín Martínez-López

**Affiliations:** 1 Servicio de Hematología y Hemoterapia. Hospital Universitario 12 de Octubre, 28041 Madrid, Spain; rayaladiaz12@gmail.com; 2 Hematological Malignancies Clinical Research Unit, CNIO, 28029 Madrid, Spain; 3 Universidad Complutense de Madrid (UCM), 28040 Madrid, Spain; 4 Centro de Investigación Biomédica en Red Cáncer (CIBERONC), 28029 Madrid, Spain

**Keywords:** minimal residual disease, next-generation sequencing, clonal evolution, hematological neoplasms, high-throughput sequencing

## Abstract

Ultra-deep next-generation sequencing has emerged in recent years as an important diagnostic tool for the detection and follow-up of tumor burden in most of the known hematopoietic malignancies. Meticulous and high-throughput methods for the lowest possible quantified disease are needed to address the deficiencies of more classical techniques. Precision-based approaches will allow us to correctly stratify each patient based on the minimal residual disease (MRD) after a treatment cycle. In this review, we consider the most prominent ways to approach next-generation sequencing methodologies to follow-up MRD in hematological neoplasms.

## 1. Introduction

In hematological malignancies, minimal residual disease (MRD) is defined as the presence of residual leukemic cells not detected by conventional morphological methods [1], and it is a powerful parameter to guide clinical management. Classically, real-time quantitative PCR (qPCR) and flow cytometric technologies have been widely utilized for MRD monitoring [2,3]. Subsequently, new assays were developed to more precisely monitor MRD, including multiparametric flow cytometry (MFC), allele-specific oligonucleotide quantitative PCR (ASO-PCR), digital PCR (dPCR), and next-generation sequencing (NGS) [4], which offer greater sensitivity than conventionally employed morphologic and cytogenetic tests. MFC provides reliable quantification of leukemic cells and is extensively available with a long clinical experience. It is nevertheless costly and difficult to standardize and has a low reproducibility. That being said, the EuroFlow group is making concerted efforts to standardize the methodology and has published two main reports regarding MRD measurement in patients with acute lymphoblastic leukemia (ALL) or multiple myeloma (MM) [5,6], and similar work is being done for chronic lymphoblastic leukemia (CLL) disease through the European Research Initiative on CLL study (ERIC) [7]. ASO-PCR is highly sensitive but laborious and time consuming, and it is not universally accessible because of its dependency on the development and validation of patient-specific primers and probes for qPCR. dPCR improves upon the sensitivity of qPCR in some validated cases and does not rely on a standard curve for sample quantification. However, the need for validation by methodological standardization programs and the challenges of having to design an experiment for each assay are limitations of the technique. By contrast, ultra-deep NGS provides clonotype quantification with specific primers (no per-patient customization is required in the case of *IGH* gene; see later) and has the benefits of high sensitivity and global applicability. In addition, NGS can be more accurate for relapse prediction in B-cell ALL [8,9] and MM [10]. Nonetheless, NGS requires a high level of expertise and complex infrastructure, although efforts are being made by several groups and sequencing companies to streamline the process. A summary of the strengths and weaknesses of the currently used methods is depicted in Table 1.

All of these methods can provide information on MRD, which can help guide risk-adapted management and predict progression-free survival (PFS) and overall survival (OS) in patients. Accordingly, MRD is now routinely incorporated into clinical algorithms for several malignant hematologic diseases including MM [11], acute myeloid leukemia (AML) [12,13,14], ALL [15], and CLL [16,17].

Focusing on molecular profiling, MRD can be studied using patient-specific oligonucleotides, which entails specific primer sets for each genomic region to be analyzed with dPCR and ASO-PCR. By contrast, NGS technology allows for the study of all the genomic regions of each pathology in a single step and is independent of the mutation site, length, and sequence. In the case of lymphoid neoplasms, analysis is usually targeted to immunoglobulin gene rearrangements in the leukemic cell, for example in MM [10,18,19], ALL [8,20], CLL [21,22], and other lymphomas [23]. In myeloid malignancies such as AML [24,25] or myelodysplastic syndromes [26], different mutations (single nucleotide variants [SNVs] or insertions/deletions [indels]) are quantified for disease monitoring.

Some of the targets utilized for MRD monitoring by NGS in hematological malignancies include:

- Repertoire sequencing, including clonal rearrangements of immunoglobulin/T-cell antigen receptor genes: *IGH* (VDJ), *IGH* (DJ), *IGK*, *TRG*, *TRD*, and *TRB* [20]. In the case of lymphocytic disorders, several markers have been tested for their utility to monitor the disease. Rearrangement of the immunoglobulin heavy chain (*IGH*) gene occurs during B-cell differentiation, making it an ideal marker for MRD monitoring owing to the fact that each neoplastic cell has the identical sequence. Several NGS-based immunoglobulin clonality assays are commercially available—for example, ClonoSEQ^TM^ [27] and LymphoTrack^TM^ [28] from Adaptive Biotechnologies^®^ and Invivoscribe^®^, respectively; but other ‘in-house’ MRD tests with a more affordable and feasible design are also used [10].
- Fusion gene quantification in lymphoid and myeloid neoplasms (e.g., *KMT2A-MLLT10*, *RUNX1*-*RUNX1T1,* and *PML*-*RARA)* [29,30].
- SNV analysis in several genes in both lymphoid and myeloid neoplasms, including *IDH1/2*, *DNM3TA*, *RUNX1*, *TET2*, *TP53,* and several indels in the *FLT3* and *NPM1* genes in AML [24,25].

**Figure 1:**
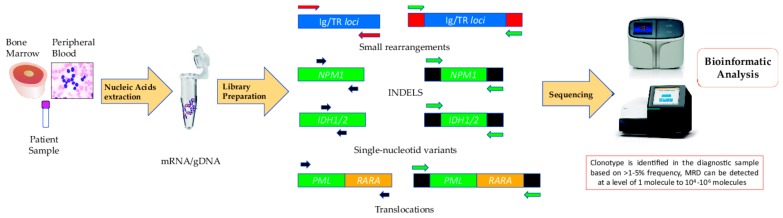
High-throughput sequencing workflow for minimal residual disease monitoring.

A summary of the NGS methods for MRD determination is provided in Table A1. A typical workflow for measuring MRD by NGS is depicted in Figure 1. RNA or DNA is extracted from peripheral blood (PB) or bone marrow (BM). The nucleic acid is then used as the input to build the corresponding libraries required for high-throughput sequencing. After correcting errors and upon appropriate alignment, MRD can be quantified.

The goal of this review is to provide a global overview of existing research on MRD quantification by NGS in different hematological pathologies, its clinical potential, and current challenges.

## 2. MRD Monitoring in Acute Myeloid Leukemia

More than half of all patients with AML who achieve negative MRD status will ultimately relapse because of the failed detection of the low levels of leukemic clones remaining during an apparent remission.

Internal tandem duplications in FMS-like tyrosine kinase-3 (*FLT3*-ITD) and mutations in nucleophosmin-1 (*NPM1*) are present in 20% and 30% of all AML patients, respectively, and they can change the evolution of the disease [31,32]. *FLT3*-ITD is variable among patients—with insertions of 3 to 400 base-pairs [33]. Although more than 16 four base-pair insertions are known in *NPM1*, 3 of them represent the bulk (95%) of the cases [34]. Accordingly, *FLT3*-ITD and *NMP1* mutations are commonly used to test new NGS platforms.

Thol et al. [35] were the first to investigate the potential of using DNA mutations found at diagnosis for MRD monitoring in AML by NGS. They sequenced *FLT3*-ITD and *NPM1* gene regions in 35 and 40 samples, respectively, from 10 patients using NGS and qPCR. The same mutations were found by both methods in 95% of the samples. They also noted the importance of the amount of DNA to increase the sensitivity of the method, and the theoretical sensitivity that could be achieved depended on the sequencing reads.

In a similar approach, Spencer et al. [36] used a multigene targeted NGS approach to sequence *FLT3.* They compared NGS with capillary electrophoresis and found that NGS detected 100% of the capillary electrophoresis-positive cases (*n* = 20) and two more cases that were not detected by this method. The authors also tested different bioinformatic pipelines and found that only Pindel [37] detected all ITD cases with an estimated variant allele frequency (VAF) of 1%.

Using NGS to assess the AML driver mutation *FLT3*-ITD, Jeffrey Miller’s group [38] used BM samples from 15 patients in complete remission (CR), with negative detection of *FLT*-ITD fragments by capillary electrophoresis and no leukemia-associated phenotype identified by 6-color MCF. This method was validated in 80 patients with *FLT*-ITD relapsed/refractory AML participating in a clinical trial of a novel FLT3 inhibitor, gilteritinib, and demonstrated a relationship between MRD measured by *FLT3*-ITD VAF and OS, with a detection threshold of 10^−4^.

At the same time, Jongen-Lavrencic and colleagues monitored MRD in 482 patients with newly-diagnosed AML, employing SNVs of various genes, with several samples mutated from 0.02 to 47% VAF [25]. They detected an average of 2.9 mutations/patient, with at least 1 mutation in 89% of the patients. MRD-positive patients had significantly shorter OS and relapse-free survival (RFS) than MRD-negative patients. Persistent mutations in *DNMT3A*, *TET2*, and *AXL1* genes were considered as MRD-positive. This study was not designed to evaluate MRD by deep-sequencing, and they did not establish the sensitivity of the sequencing by diluting a mutated sample.

Several other genes such as *ASXL1*, *CEBPA*, *DNMT3A*, *FLT3*, *NPM1*, *SFSR2,* and *TET2* have been analyzed to demonstrate that mutation clearance is associated with significantly better event-free survival, OS, RFS, or less risk of relapse [39,40].

An error-corrected NGS MRD approach was reported by Thol and collaborators with 116 AML patients undergoing allogeneic hematopoietic cell transplant (allo-HCT) in CR. MRD positivity (VAF <5%) stratified the patients into a higher cumulative incidence of relapse and lower OS. In addition, MRD positivity was an independent negative predictor of *FLT3*-ITD and *NPM1* status at diagnosis and to TP53-KRAS mutation status and conditioning regimen [14].

In a recent study by Onecha and collaborators, MRD was measured with *NPM1* and SNVs of *IDH1/2* and *DNMT3A* in 106 samples from 63 patients [24]. The OCI-AML3 cell line was used to determine the limit of detection by NGS and dPCR, which was found to be 0.001% by NGS for the *NPM1*-insertion and 0.01% for SNV mutations, which is one-to-two orders of magnitude lower than that for dPCR. The MRD status determined by NGS had prognostic value in AML patients. Using receiver operating characteristic curves after induction and consolidation, cut-offs of 0.1% and 0.025%, respectively, were determined. Patients who were MRD-positive after induction (≥0.1%, *n* = 35) were associated with significantly shorter OS but non-significant disease-free survival (DSF). In the case of MRD-positive patients after consolidation (≥0.025%, *n* = 28), both curves showed significance between the two compared groups. Accordingly, MRD determination by this NGS method improved the prediction of the outcome of AML patients over dPCR.

Concurrent with the publication of the Onecha study, Malmberg and colleagues reported a method whereby MRD was quantified by deep sequencing of SNVs [29]. The limit of detection of the technique was estimated from 15 mutated samples, with a VAF outcome of 0.02%. They also demonstrated that dual indexing of the samples reduced barcode contamination. Thirty-four BM samples from six children were analyzed, and MRD by deep sequencing was concordant with MFC and had higher sensitivity. In addition, they compared MRD for three cases with RUNX1-RUNX1T1 and KMT2A-MLLT10 rearrangements, resulting in 13 out of 14 concordant MRD assignments (eight positives and five negatives). The same results were obtained with PB samples, confirming the clinical applicability of this specimen when VAF in BM exceeds 0.1%.

In summary, the most important targets for monitoring MRD in AML are *FLT3*-ITD and *NPM1*. The input DNA amount ranges from 100 to 700 ng, and sequencing can be performed by both Illumina and Thermo Fisher platforms. The sensitivity reaches 10^−5^, which is equivalent to a VAF of 0.001%. The specimen used for measuring MRD can also be PB or BM when handling VAF above 0.1%.

There is no evidence to date that altering clinical management upon positive MRD by NGS (after induction or at relapse) modifies the outcome of patients with AML (with the exception of post-allo-HCT and the PML-RARA fusion oncoprotein in acute promyelocytic leukemia). Despite this, MRD measurement by NGS in AML has been widely reported in the last three years [14,24,25,29,38,39], but clinical validation of this promising technology is still needed.

## 3. MRD Monitoring in Lymphoid Malignancies

### 3.1. Acute Lymphoblastic Leukemia

Acute B-cell lymphoblastic leukemia is caused by a group of genetic alterations that result in the uncontrolled proliferation of lymphoid progenitor cells in BM, PB, and other extramedullary sites, with approximately four-fifths of all ALL cases occurring in children. MRD monitoring following ALL therapy has increased in prognostic relevance in recent years. Clonal *IGH* rearrangement is an effective follow-up marker, mainly in ALL lacking the *Philadelphia* chromosome-translocation, due to its high positivity rate. Other markers for MRD monitoring in T-cells include the variable regions of the T-cell receptor (TCR), such as the loci *TCRB*, *TCRD*, and *TCRG*, which could be rearranged in lymphoid malignancies.

In this scenario, a clonotype is defined as the number of sequences found in a patient’s repertoire that are identically present above a threshold percentage. This applies in both TCR/Ig loci, and typically the cutoff point is ~5%.

The first NGS studies using B-cells to measure MRD was performed by Gawad et al. [41], who studied the *IGH* repertoire in diagnostic samples of 51 children newly diagnosed with B-ALL. Eighty-four percent of the cases showed clonal rearrangements. The authors demonstrated the association between the aberrant sequences and the leukemic precursor B-cells, and this occasionally occurred only at one of the two alleles of the *IGH* loci.

Three technical validation approaches for MRD monitoring in ALL have been reported thus far [27,42,43]. Faham’s group defined a new high-throughput sequencing (HTS) method (termed LymphoSIGHT^TM^) capable of amplifying and identifying all clonal gene rearrangements at the moment of diagnosis and allowing monitoring of clonal evolution during treatment [27]. After discarding inherent errors during the analysis of the generated clonotypes, clonal rearrangements were detected in several immune cell receptor genes in the BM of 105 patients. The methodology was compared with MFC and ASO-PCR. Regarding NGS versus MFC, 90% of the samples (95/105) gave concordant results (positive or negative MRD). When ASO-PCR and NGS techniques were compared, 102 out of 106 evaluated samples were concordant; three of them were positive only by NGS and one of them was undetectable by NGS but detectable by ASO-PCR. Along the same line, a procedure to deep-sequence the *Ig/TCR* genes was reported by Salson and collaborators [42], who analyzed the clonality of 58 samples from 11 pediatric patients with B- and T-ALL. The output data was examined with the Vidjil web application, which was used to correct sequencing errors [44]. Comparing NGS with qPCR, the authors found that the results were in accord in 22 out of the 32 samples, and NGS detected five additional follow-up samples with a MRD below 10^−4^, which were negative by qPCR. Vidjil software was also used by Ferret and coworkers with diagnostic and relapsed samples in 34 pediatric patients [43]; with this pipeline, the homopolymer errors sequences—inherent to Ion Torrent sequencing—were discarded, and the V(D)J recombination consensus sequences were correctly designated.

Evaluation of residual disease by massive sequencing was also useful to stratify a group of patients with lower leukemia-free survival and RFS [45,46]. Sekiya et al. monitored MRD in a cohort of 79 patients at different stages of treatment and found inferior leukemia-free survival in patients with positive MRD after 80 days, 4–5 months, or 2 years [45]. Sala Torra and coworkers analyzed MRD by NGS in 153 samples from 32 patients and concluded that the tumor burden was lower in PB than BM. Furthermore, patients with negative MRD had a 5-year RFS of >80% [46].

MRD measured by NGS was also crucial to predict future recurrence in three reports [8,20,47]. Theunissen et al. sequenced 42 paired children samples at diagnosis and relapse [20]. They showed that it was possible to follow-up 97% of patients until relapse, with clonotype repertoires by NGS with five different targets. In a second report, Cheng et al. [8] sequenced 122 samples from 30 B-ALL patients using the commercial kit LymphoTrack^®^. The dominant tumor clone was identified by the quantification of 5% of the total reads per sample. With a sensitivity of 10^−6^, they detected 22 samples that were MRD-negative by MFC. Finally, Pulsipher and coworkers predicted relapse and survival more precisely by NGS in comparison with MFC in a cohort of 56 patients that underwent HCT [47]. The precision was more evident at day 30 post-HCT, where MRD-positive cases were 67% versus 35% by MFC. In addition, a subset of MRD-negative patients was stratified as good-risk; this was useful to diminish the intensity of further treatments.

The TCR locus was also analyzed by NGS, specifically *TCRB* and *TCRG,* to track MRD in T-ALL [48]. Thirty-one out of 43 cases (72%) presented a monoclonal sequence at diagnosis. Moreover, MRD by NGS was more sensitive than MFC, and 32% of the MRD subset of patients was detected only by NGS.

An in-house developed NGS methodology for Ig rearrangements was established by Batram and colleagues to identify clonal differences between paired BM and central nervous system (CNS) samples, concluding that same clones present at BM can disseminate to the CNS [49] and thus can be monitored. In a similar line, tumor burden status in *BCR-ABL1*-positive patients with ALL was analyzed [50], and the allelic frequency of the kinase domain mutations in *BCR-ABL1* in cerebrospinal fluid (CSF) blasts was useful to predict CNS relapse. These results confirmed the importance of analyzing CSF blasts in the case of suspicion of CNS relapse, with the aim of detecting independent mutations that could be exclusive to this site [50].

In summary, the main target for monitoring MRD in ALL is the *IGH* gene. The input DNA amount tested reached up to 5 µg, but this would be necessary only in a few follow-up cases, and for routine analysis, only a few hundred nanograms of DNA would be needed. For ALL disease, the sensitivity reaches 10^−6^, which equates to one leukemic cell in one million healthy leukocytes. The specimen sample used for measuring MRD has been mainly BM, although two reports have also tested PB with similar results [20,46]. The strong correlation between MRD and risk of relapse and the prognostic significance of MRD measurements during and after induction therapy has already been acknowledged in the pediatric and adult ALL population [15]. The greater sensitivity achieved by NGS means that this technique will ultimately replace MFC to become the gold standard for the follow-up of ALL patients along their treatments.

### 3.2. Chronic Lymphocytic Leukemia

CLL is a clonal B cell disease that infiltrates the BM, PB, and often lymph nodes, and has a low-level expression of surface immunoglobulins. Those with high-risk disease are frequently treated with allo-HCT after relapse or refractory response. Because of this, monitoring of the malignancy during treatment is essential.

The first report on high-throughput sequencing of the VDJ segment on the *IGH* gene was described by Logan and collaborators [51], who provided CLL clonotype quantification with two different consensus sets of non-specific patient primers [52], improving sensitivity and applicability. The experiments were performed by pyrosequencing, reaching a sensitivity of 10^−5^. Sequencing was carried out on samples from six CLL patients, and the authors proposed a significant role of MRD in CLL patient handling following allo-HCT. Although HTS results had no correlation in comparison with flow cytometry (*r* = 0.49), the correlation was much greater if it was compared with ASO-PCR (*r* = 0.85). Furthermore, this method allowed the authors to characterize *IGH* repertoire reconstitution after allo-HCT. These findings would be the starting point for monitoring different types of pathologies with rearranged immunoglobulin genes.

Logan’s group also studied MRD in CLL using the LymphoSIGHT^TM^ method [21], which was previously developed by Faham et al. [27] for ALL. After sequencing, a home-made pipeline was created to remove artifacts and quantify MRD from the *IGH* gene. They studied more than 400 samples from 40 patients who underwent reduced-intensity allo-HCT. MRD-positive patients had a DFS of 86% versus 20% in MRD-negative patients. The calculated MRD was useful to predict relapse in several post-HCT milestones.

Stamatopoulos and collaborators have recently sequenced the IgHV locus of 227 + 270 patients in two separate populations [53], one using samples from patients at diagnosis, who would receive or not treatment, and another population belonging to the AdMIRe and ARCTIC clinical trial (schemes based on fludarabine, cyclophosphamide, and rituximab). They applied a commercial methodology with IGH LEADER and consensus JH primers. With this methodology, the authors found 24% of patients with multiple productive rearrangements (66/270) and created five categories based on the number and mutational status of the clones, from multiple mutated clones to multiple unmutated clones. This classification was associated with different treatment-free survival. The subclonal rearrangements obtained are expected to contribute to the biological heterogeneity of CLL and suggest initial pre-leukemic events that foresee IgHV rearrangements.

The design of a custom gene panel intended for clinical utilization in CLL is ongoing [54,55]. Despite the large increase in the number of genes with prognostic value, the only biomarker that currently influences the treatment strategy is TP53 [56]. Accordingly, Rossi et al. reported a method to determine the clinical impact of minimal *TP53* mutated clones in CLL by ultra-deep pyrosequencing. Patients harboring small TP53-mutated subclones showed the same poor survival (hazard ratio = 2.01; *p* = 0.025) as that of patients carrying clonal TP53 lesions. With this method, it was possible to detect small subclones in early phases of the disease that could anticipate a possible relapse and the development of the chemorefractory phenotype.

As for ALL, the main target for the quantification of MRD in CLL is *IGH*. DNA has been isolated from PB mononuclear cells in most of the cases. The highest sensitivity achieved was 10^−6^, similar to that for ALL. Overall, there is scientific evidence to assume that NGS will be used to assess deep response and will become an important method to assess survival and for the approval of new drugs in diseases like CLL [57,58,59,60,61]. Although MRD quantification by MFC is quite standardized [62], the newly developed NGS technology will very likely compete in the future for the measurement of MRD not only in CLL disease but also in all B-cell malignancies.

### 3.3. Multiple Myeloma

In recent years, patients with MM have experienced improved CR rates and increased survival; however, many patients relapse because of undetectable MRD. MRD detection in MM using MCF and PCR-based methods has been broadly studied over the past twenty years [23,63,64,65], but novel methodologies such as dPCR or high-throughput sequencing is slowly being introduced.

The first approaches to measure MRD by NGS in patients with MM were developed by the groups of Martinez-Lopez and Vij and their collaborators [66,67]. Both employed the LymphoSIGHT^TM^ methodology [27] previously applied to other B-cell malignancies [21]. Vij and coworkers analyzed 60 paired BM and PB samples—47 from patients and 13 of commercial origin. The method was established with the *IGH-VDJ*, *IGH-DJ*, and *IGK* genes. Forty-eight of the 60 samples showed high-frequency rearrangements in at least one of the three markers analyzed, with *IGH*-*VDJ* being the most instructive (44/60, 73%). The PB myeloma clone levels were ~100-fold lower than in the corresponding BM samples. This study did not report the use of MRD in clinical studies. Although detection of myeloma clone levels in PB samples was 2-log lower than in BM samples, the authors detected myeloma cells in most of the patients (44/46, 96%) when RNA was employed as starting material instead of DNA, which is less sensitive.

In contrast to the study by Vij et al. [67], Martinez-Lopez and colleagues demonstrated the prognostic value of MRD by NGS to stratify patients with MM [66]. They collected BM samples from 133 patients who achieved at least very good partial response after front-line treatment. The samples were collected after induction, or after induction and hematopoietic stem cell transplantation, depending on the age of the patients. The *IGH*-VDJ locus was the most frequent rearrangement found. MRD levels of 10^−5^ or higher were identified in 80 out of 110 of the follow-up samples (73%). MRD levels lower than 10^−5^ were associated with significantly longer OS than patients with positive MRD (>0.1%). The prognostic significance was maintained when the patients were separated by age-range (young/elderly). Fifty-eight percent of the patients in CR status had MRD levels higher than 0.001%, but patients who were MRD-negative had a significantly longer time to tumor progression compared with patients in CR who were MRD-positive by sequencing. The sensitivity of the method was 1 to 2 logs higher than that achieved by MFC.

Avet-Loiseau’s group monitored MRD in BM samples by NGS in two randomized, controlled phase 3 clinical trials, concluding that the addition of daratumumab to standard of care treatments resulted in significantly higher MRD negative rates in all three investigated cutoff thresholds (10^−4^, 10^−5^, and 10^−6^) [68].

A novel in-house methodology for monitoring MRD in MM patients was developed by Martinez-Lopez and collaborators [10]. They sequenced 73 BM samples from patients enrolled in a phase 2 trial for newly diagnosed elderly patients. A clonotype was detected in 71 out of the 73 patients (97%). The proportion of MRD-negative patients was higher after 18 cycles of treatment than after nine cycles, confirming that prolonged treatment time improves the molecular response. Comparing MRD-positive and MRD-negative patients, OS and DFS was significantly longer (*p* ≤ 0.05) for patients that obtained negative MRD status.

A new study for measuring MRD in MM has been recently published by Perrot and collaborators [69]. They sequenced BM samples of patients with a commercial test using immunoglobulin gene-specific primers. MRD status was assessed in 407 samples at the start or after completing maintenance therapy; 138 of the samples belonged to the same patient. Data were analyzed to evaluate the role of HCT in newly diagnosed MM patients treated or not treated with lenalidomide, bortezomib, and dexamethasone. At the points of evaluation of the disease beginning and end of maintenance therapy, MRD was a prognostic factor for PFS and OS. Patients with MRD of <0.0001% had a higher probability of prolonged PFS than did patients with detectable MRD, regardless of treatment, cytogenetic risk profile, or disease stage at diagnosis according to the International Staging System.

The introduction of this new technology for measuring MRD in MM has promoted the definition of new criteria for MRD assessment by the International Myeloma Working Group [11]. The last three studies demonstrate the capability of NGS for evaluating MRD status as a prognostic biomarker in MM, succeeding in guiding new treatment strategies in ongoing clinical trials. Moreover, MRD measurement has been approved by the FDA as a surrogate marker for new drug approval in clinical trials.

## 4. Current Challenges

NGS is a promising tool for MRD detection and could be the new gold standard for several hematological diseases in the near future. However, the workflow needs to be standardized before its implementation into routine clinical practice [70]. MRD levels in ALL tend to be 1–3 logs greater in BM than in PB [9,71], and thus the optimal biological sample to measure MRD could be BM for patients with ALL or MM. For other diseases such as AML, a PB specimen might be optimal, depending on the chemotherapy cycle, but BM is also valid. More work is needed to better decide the usage of PB or BM for measuring MRD by NGS [12].

It is well known that DNA amount is crucial to attain good sensitivity, and Cheng and coworkers recommended 1–5 μg DNA as the input amount [8]. It is, however, very difficult to routinely obtain these amounts of DNA for all the follow-up samples, so it would be advisable to have around 500 ng for the diagnosis and to increase the amount progressively at each treatment point to reach 6 μg [72]. These amounts are only suggestions based on prior experimental procedures, but until there is a consensus of use, each amount of starting DNA must be optimized for each disease and procedure.

The use of DNA allows clinicians to obtain tumor burden information, and the comparison with mutated genomic DNA is feasible [24]; RNA offers information about the variety of gene expression and it is variable among cells. In addition, RNA might be the most appropriate template when monitoring a disease caused by fusion transcripts [30].

Although a sensitivity of 10^−7^ can be theoretically achieved, this depends on the number of sequencing reads [35], which in turn depends on the starting DNA input amount. This is a major limitation when analyzing samples after myelotoxic treatment, and more than 10 μg of DNA would be necessary to accomplish the protocol.

Sensitivity versus specificity is an important consideration to address in MRD monitoring approaches by NGS [70,73]. While error-corrected sequencing (ECS) and other bioinformatic approaches help in removing background error, these tools are not yet perfect. As a result, much of the sensitivity seen by NGS will presumably have to be sacrificed for specificity. Cheng and collaborators demonstrated a specificity of 100% measuring MRD for 16 B-ALL samples (diagnostic and follow-up) and 20 unrelated B-ALL samples. The leukemia-specific IGH clonotypes were only detected in B-ALL samples with a sensitivity of ~2 × 10^−6^ [8].

Because NGS introduces errors into DNA sequences as a consequence of initial DNA amplification and NGS sequencing by itself, the incorporation of unique molecular index (UMI) sequences can be used to redress this problem. A UMI is a short sequence (8–16 nucleotides) that is specific to a molecule and is generated by permutations of a string of randomized nucleotides [74]. To distinguish false-positive mutations from true-mutations, raw sequencing reads are grouped according the UMI; artifacts from sequencing are not expected at the same position in sequences with the same UMI [75]. Several methods for introducing UMI sequences have been created [74]. The use of UMI sequences to discriminate real variants from protocol artifacts applied to clinical samples in patients with AML and myeloid dysplastic syndromes was first reported by Young and coworkers [76]. Thol et al. also reported the use of UMI sequences during library preparation and duplex sequencing data analysis for ECS [77].

Several ways to reduce the sequencing error rate include the use of a proofreading polymerase for PCR, the use of low number of cycles at initial PCR, trying to sidestep the same patient identifiers on consecutive NGS runs, and creating a standardized bioinformatic pipeline ready to correct sequencing errors [14].

Dillon and collaborators have recently described MRD measured by high-sensitivity RNA-sequencing with a limit of detection of 10^−5^ in a subset of AML patients [30]. The targets were those newly approved by European Leukemia Net for measuring MRD in AML, such as NPM1 or PML-RARA, among others. With this work, a new field has now emerged with the advantages offered by working with RNA, such as the monitoring of fusion transcripts and the use of UMI sequences, which allows the absolute quantification of the levels of each transcript.

Importantly, spike-in calibrators and quality controls are mandatory to guarantee the veracity of the results for both assay validation and cross-comparison between studies, with the aim to make clinical decisions safely [78,79].

Along this line, the EuroClonality-NGS consortium was established with the aim to set the bases for the standardization and validation of MRD determination with the final goal to apply this technology to clinical routine [80]. To do this, it is necessary to bring together knowledge on molecular biology, hematology, and bioinformatics, with people with a high degree of expertise in these areas.

Given the great applicability and versatility of NGS and the multitude of target markers in the case of AML, an increase in the use of the technique is expected once it is standardized. The correlations with other techniques such as ASO-PCR, MFC, or qPCR are good [27,28,42,51], but many clinical validation studies are still necessary for each different pathology. At this moment, the number of centers capable of performing MRD detection by NGS is low; this is obviously a limitation for the development of the technique, but this will change in the coming years.

The era of increasingly personalized medicine inspires us to combine multidisciplinary efforts so that in the near future, the measurement of MRD by NGS will be a routine procedure.

## Figures and Tables

**Table 1:** Summary of advantages and disadvantages for measuring minimal residual disease (MRD) with the available technology.

Methodology	Strengths	Weaknesses
Multiparameter Flow Cytometry	Fast; Absolute Quantification; Information at a cellular level; Wide availability	Variable antigen expression could lead to false negative results; High grade of expertise needed; Medium sensitivity with less than 8-colours
Allele-Specific Oligonucleotide PCR	High sensitivity	Time-consuming in the design of patient-specific primers; Requirement for optimal DNA quality and quantity
Digital PCR	Absolute quantification; High sensitivity; Avoids PCR inhibitors due to compartmentalization of target sequences	Lack of standardization No possibility to find new variants Allele-specific design
Next-Generation Sequencing or High-Throughput Sequencing	High Sensitivity (>10^−6^); Patient-specific primers not necessary; Versatility	Lack of standardization; High degree of bioinformatics expertise; Expensive

## Abbreviations

ALL	Acute Lymphoblastic Leukemia
AML	Acute Myeloid Leukemia
ASO-PCR	Allele-Specific Oligonucleotide PCR
BM	Bone Marrow
CLL	Chronic Lymphocytic Leukemia
CNS	Central Nervous System
CR	Complete Remission
CSF	Cerebro-Spinal Fluid
DFS	Disease-Free Survival
ITD	Internal Tandem Repeats
MFC	Multiparametric Flow Cytometry
MM	Multiple Myeloma
MRD	Minimal Residual Disease
NGS	Next-Generation Sequencing
OS	Overall Survival
PB	Peripheral Blood
PFS	Progression-Free Survival
qPCR	Quantitative Polymerase Chain Reaction
RFS	Relapse-Free Survival
SNV	Single Nucleotide Variation
UMI	Unique Molecular Index
VAF	Variant Allele Frequency

## Appendix A

**Table A1:** High-throughput methods of minimal residual disease determination in hematological malignancies.

Hematological Malignancy	Authors	NGS Methodology-Equipment	Target	Sensitivity	Year
AML	Thol et al.	Pyrosequencing 454 Junior	*FLT3*-ITD *NPM1*	NE	2012
AML	Spencer et al.	Sure Select- Illumina-HiSeq	*FLT3*-ITD	NE	2013
AML	Klco et al.	Ion Torrent-PGM	xGen^®^ AML Cancer Panel	NE	2015
AML	Jongen-Lavrencic et al.	Illumina-MiSeq	TruSight^®^ Myeloid Sequencing Panel	NE	2018
AML	Levis et al.	Illumina-MiSeq	*FLT3*-ITD	10^−6^	2018
AML	Morita et al.	Illumina-HiSeq 2000	*NPM1* *DNMT3A* *FLT3-ITD*	NE	2018
AML	Thol. et al.	Illumina-MiSeq	*IDH1 ** *DNMT3A*	5 × 10^−5^	2018
AML	Onecha et al.	Ion Torrent-Proton PI	*NPM1 ** *IDH1* *IDH2*	5 × 10^−5^	2019
AML	Malmberg et al.	Illumina-MiSeq	TruSight Myeloid Sequencing Panel *RUNX1*/*RUNX1T1* *KMT2A*/*MLLT10*	NE	2019
CLL	Logan et al.	Pyrosequencing 454 Junior	*IGH*(VDJ)	10^−5^	2011
CLL	Logan et al.	Illumina	*IGH*(DJ)	10^−6^	2013
CLL	Rossi et al.	Pyrosequencing 454 Junior	*TP53*	NE	2014
CLL	Stamatopoulos et al.	Illumina-MiSeq	*IGH* LEADER (VDJ)	NE	2017
ALL	Wu et al.	Illumina-HiSeq	*TRB ** *TRG*	10^−5^	2011
ALL	Gawad et al.	Illumina-MiSeq	*IGH*(VDJ)	NE	2012
ALL	Faham et al.	Illumina-MiSeq	*IGH*(VDJ) * *IGH*(DJ) * *TRB* *TRD* *TRG*	10^−6^	2012
ALL	Wu et al.	ClonoSEQ assay-Illumina	*IGH*(VDJ) *IGH*(DJ)	10^−6^	2014
ALL	Pulsipher et al.	Illumina-HiSeq	*IGH*(VDJ)	10^−7^	2015
ALL	Ferret et al.	Ion Torrent-PGM	*IGH*(VDJ) *IGK* *TRG* *TRD*	NE	2016
ALL	Sekiya et al.	Illumina-MiSeq	*IGH(VDJ) ** *TRG*	10^−6^	2016
ALL	Sala Torra et al.	ClonoSEQ assay-Illumina	*IGH*(VDJ) *IGH*(DJ)	NE	2017
ALL	Salson et al.	Ion Torrent- PGM	*IGH*(VDJ) * *TRG*	2 × 10^−5^	2017
ALL	Cheng et al.	Illumina-MiSeq	*IGH(VDJ)*	10^−6^	2018
ALL	Theunissen et al.	Illumina-MiSeq	*IGH*(VDJ) *IGK* *TRG* *TRD* *TRB*	NE	2018
ALL-CNS	Sanchez et al.	Ion Torrent-S5 XL	KD of *BCR*-*ABL1*	NE	2017
ALL-CNS	Bartram et al.	Illumina-MiSeq	*IGH*(VDJ)	NE	2018
MM	Vij et al.	Illumina-MiSeq	*IGH*(VDJ) *IGH*(DJ) *IGK*	NE	2014
MM	Martinez-Lopez et al.	Illumina-MiSeq	*IGH*(VDJ)	10^−5^	2014
MM	Avet-Loiseau et al.	ClonoSEQ assay-Illumina	*IGH*(VDJ) *IGH*(DJ)	NE	2016
MM	Martinez-Lopez et al.	Ion Torrent-S5 XL Illumina-MiSeq	*IGH*(VDJ) * *IGH*(DJ) * *IGK**	10^−5^	2017
MM	Perrot et al.	Illumina-MiSeq	*IGH*(VDJ)	10^−6^	2018

ALL—Acute Lymphoblastic Leukemia; AML—Acute Myeloid Leukemia; CNS—Central Nervous System; CLL—Chronic Lymphocytic Leukemia; KD—Kinase Domain; MM- Multiple Myeloma; NE—Not evaluated; NGS—Next-Generation Sequencing. The target marked with an asterisk (*) was used to determine sensitivity.
